# Hydrophobia—Prevention and Cure

**Published:** 1884-06

**Authors:** 


### Hydrophobia—Prevention and Cure.

Dr. M. L. Pasteur, the celebrated chemist, is said to have discovered an absolute cure, or rather antidote, for hydrophobia. In an interview he says:

"Cauterization of the wound immediately after the bite, as is well known, has been more or less effective, but from to-day anybody bitten by a mad dog has only to present himself at the laboratory of the Ecole Normale, and by inoculation I will make him completely insusceptible to the effects of hydrophobia, even if bitten subsequently by any number of mad dogs. I have been devoting the last four years to this subject. I found out in the first place that the virus rabique loses its intensity by transmission to certain animals and increases its intensity by transmission to other animals. With the rabbit, for instance, the virus rabique increases; with the monkey it decreases. My method was as follows: I took the virus direct from the brain of a dog that had died from acute hydrophobia. With this virus I inoculated a monkey. The monkey died. Then, with the virus—already weakened in intensity—taken from this monkey I inoculated a second monkey. Then with the virus taken from the second monkey, I inoculated a third monkey, and so on, until I obtained a virus so weak as to be almost harmless. Then, with this almost harmless virus, I inoculated a rabbit, the virus being at once increased in intensity. Then, with the virus from the first rabbit, I inoculated a second rabbit, and there was another increase in the intensity of the virus. Then, with the virus of the second rabbit, I inoculated a third rabbit, then a fourth, until the virus had regained its maximum intensity. Thus I obtained a virus of different degrees of power. I then took a dog and inoculated him first with the weakest virus from the rabbit, then with the virus from the second rabbit, and finally with the rabbit virus to maximum intensity. After a few days more I inoculated the dog with virus taken directly from the brain of a dog that had just died of acute madness. The dog upon which I had experimented proved completely insusceptible to hydrophobia. The experiment was frequently repeated, always with the same successful result.

"But my discovery does not end here, I took two dogs and inoculated them both with virus taken directly from a dog that had just died of acute hydrophobia. I let one of the dogs thus inoculated alone, and he died of acute hydrophobia. I subjected the second dog to my treatment, giving him the weakest and ending with the strongest. This second dog was completely cured, or rather became completely insusceptible to hydrophobia.

M. Pasteur then went to a kennel and caressed a dog that had undergone this latter operation. "Vayezo," said M. Pasteur, "comme il est bein gentil." Whoever gets bitten by a mad dog has only to submit to my three little inoculations, and he need not have the slightest fear of hydrophobia."

